# Genome-scale resources for *Thermoanaerobacterium saccharolyticum*

**DOI:** 10.1186/s12918-015-0159-x

**Published:** 2015-06-26

**Authors:** Devin H Currie, Babu Raman, Christopher M Gowen, Timothy J Tschaplinski, Miriam L Land, Steven D Brown, Sean F Covalla, Dawn M Klingeman, Zamin K Yang, Nancy L Engle, Courtney M Johnson, Miguel Rodriguez, A Joe Shaw, William R Kenealy, Lee R Lynd, Stephen S Fong, Jonathan R Mielenz, Brian H Davison, David A Hogsett, Christopher D Herring

**Affiliations:** Mascoma Corporation, 67 Etna Rd, 03766 Lebanon, NH USA; BioEnergy Science Center, Oak Ridge National Laboratory, P.O. Box 2008, Oak Ridge, TN 37831 USA; Dow AgroSciences, 9330 Zionsville Road, Indianapolis, IN 46268 USA; Chemical and Life Science Engineering, Virginia Commonwealth University, P.O. Box 843028, Richmond, Virginia 23284 USA; Centre for Applied Bioscience and Bioengineering, Department of Chemical Engineering and Applied Chemistry, University of Toronto, Toronto, Canada; Novogy Inc, Cambridge, MA 02138 USA; Thayer School of Engineering, Dartmouth College, 14 Engineering Drive, Hanover, NH 03755 USA

## Abstract

**Background:**

*Thermoanaerobacterium saccharolyticum* is a hemicellulose-degrading thermophilic anaerobe that was previously engineered to produce ethanol at high yield. A major project was undertaken to develop this organism into an industrial biocatalyst, but the lack of genome information and resources were recognized early on as a key limitation.

**Results:**

Here we present a set of genome-scale resources to enable the systems level investigation and development of this potentially important industrial organism. Resources include a complete genome sequence for strain JW/SL-YS485, a genome-scale reconstruction of metabolism, tiled microarray data showing transcription units, mRNA expression data from 71 different growth conditions or timepoints and GC/MS-based metabolite analysis data from 42 different conditions or timepoints. Growth conditions include hemicellulose hydrolysate, the inhibitors HMF, furfural, diamide, and ethanol, as well as high levels of cellulose, xylose, cellobiose or maltodextrin. The genome consists of a 2.7 Mbp chromosome and a 110 Kbp megaplasmid. An active prophage was also detected, and the expression levels of CRISPR genes were observed to increase in association with those of the phage. Hemicellulose hydrolysate elicited a response of carbohydrate transport and catabolism genes, as well as poorly characterized genes suggesting a redox challenge. In some conditions, a time series of combined transcription and metabolite measurements were made to allow careful study of microbial physiology under process conditions. As a demonstration of the potential utility of the metabolic reconstruction, the OptKnock algorithm was used to predict a set of gene knockouts that maximize growth-coupled ethanol production. The predictions validated intuitive strain designs and matched previous experimental results.

**Conclusion:**

These data will be a useful asset for efforts to develop *T. saccharolyticum* for efficient industrial production of biofuels. The resources presented herein may also be useful on a comparative basis for development of other lignocellulose degrading microbes, such as *Clostridium thermocellum*.

**Electronic supplementary material:**

The online version of this article (doi:10.1186/s12918-015-0159-x) contains supplementary material, which is available to authorized users.

## Background

Whether biomass-derived fuels play a major role in the world’s energy future depends on the development of technology to produce them at a cost that is competitive with petroleum and other alternatives [1]. Fermentation of lignocellulose (a mix of polymeric substances which are composed of a variety of sugars that in turn provide the primary structure to plants) is an attractive approach to fuel production given that plants are the most common raw organic feedstock on earth [2,3]. The development of better fermenting organisms could achieve much of the necessary cost reductions [4-6]. This represents an opportunity to apply recent advances in metabolic engineering and systems biology to a problem of major importance: the need for carbon-neutral fuels [7].

The thermophilic anaerobic bacteria include species with natural abilities to digest and ferment the polysaccharides that make up the bulk of lignocellulosic biomass [8,9]. Unfortunately, the lack of information and resources for these organisms has hindered their development. *Thermoanaerobacterium saccharolyticum* is a Gram positive, low G + C bacterium in the phylogenetic class “*Clostridia*” [10]. Members of the genus *Thermoanaerobacterium* are thermophilic, rod shaped, chemoorganotrophic and able to reduce thiosulfate to elemental sulfur. The species *T. saccharolyticum* can ferment a wide array of carbohydrates, such as starch, xylan, glucose, cellobiose, xylose, arabinose, mannose, and galactose, but cannot degrade crystalline cellulose [10]. Most sugars are fermented to ethanol, acetic acid, lactic acid, carbon dioxide and hydrogen [4]. *T. saccharolyticum* has a temperature range of 45–70°C, and pH range between 4.5-7.0. The formation of endospores has not been observed in this species as they have in the related genus *Clostridium*.

A variety of thermophilic enzymes of industrial utility have been isolated from *T. saccharolyticum*, including endoxylanase, beta-xylosidase, amylopullanase and glucuronidase [11-17]. A system for genetic manipulation of *T. saccharolyticum* was first described by Mai et al. [18], which has been improved by the discovery of natural competence [19], and the development of methods for making unmarked mutations with negatively selectable markers [20]. The genes for lactate dehydrogenase, phosphate transacetylase and acetate kinase were knocked out using these methods [4,20]. The result was a strain that produces ethanol at greater than 90% of theoretical yield, comparable to other ethanologens such as yeast, *E. coli* or *Z. mobilis* [21,22]. The advantages that *T. saccharolyticum* has over these other biocatalysts are its elevated growth temperature (matching the temperature optimum of many cellulases [21,23]), and its ability to hydrolyze hemicellulose and co-ferment the major sugars present in lignocellulose [24].

Cellulosic biomass from plants is prepared for hydrolysis and fermentation by various forms of pretreatment in order to expose the cellulose fibers and reduce particle size, though inhibitory compounds, such as furfural and hydroxymethyl furfural (HMF) are generated in the process [25]. Cost effective ethanol production requires ethanol concentrations > 40 g/L, which necessitates that substrates, and by the same token their inhibitors, be present at fairly high concentrations. The ability to reduce costs by increasing levels of pretreated substrate is limited by the levels of inhibitors in the fermentation. While there is great potential to reduce costs by developing organisms with greater tolerance to inhibitors, little is known about the effects of these compounds on microbial physiology. One of the goals of this project was to generate information about the effects of specific inhibitors and complex inhibitor extracts from pretreated material. The project was undertaken as part of a larger project to develop *T. saccharolyticum* for fermentation of pretreated hardwood [26].

Another goal was to compare the genome of *T. saccharolyticum* to the genomes of other bacteria potentially important for biofuel production, including *Clostridium thermocellum*, an organism highly specialized for the hydrolysis of cellulose and the focus of other OMICs and systems biology efforts. This work supplements the knowledge about both these important organisms and presents a comprehensive resource for further investigation.

## Results and discussion

### Genome sequencing

As the sequence was being generated, there were early indications that contig 2 was in fact a megaplasmid. Furthermore, early draft sequences showed that the ends of contig 2 overlapped. When PCR primers were designed at the ends of contig 2 facing outwards, they amplified a product consistent with a circular DNA molecule. The gene *Tsac_2822* on the putative megaplasmid encodes a RepB DNA replication protein with high similarity to replication proteins from a number of bacterial megaplasmids. These include: *C. botulinum* plasmid pCLI (BLAST E-value: 1e-64), *B. methanolicus* plasmid pBM19 (BLAST E-value: 9e-58), and *B. weihenstephanensis* plasmid pBWB402 (BLAST E-value: 3e-56). Contig 2 was poorly represented in the initial Sanger sequence data and was observed to be completely absent in strain ALK2; its loss as a complete unit further supporting its identification as an extra-chromosomal unit [4].

The genome contains 39 ORFs predicted to have transposase function, with 12 of these concentrated in a 50 kbp region. The tool Prophage Finder [27] was used to identify two regions containing genes with similarity to known phage genes using the software’s strict search settings (E-value cut off = 0.001). These two regions are 36 kbp and 42 kbp, located between ORFs Tsac_2404 – Tsac_2458 in contig 1 and between ORFs Tsac_2829-Tsac_2885 in contig 3 (the later listed under a separate accession number in GenBank, CP003186). Close examination of individual reads of CP003186 showed that some proceeded from the phage into contig 1 near position 2,009,359, suggesting a phage integration site. Contig 1 reads showed that in some, but not all of them the phage was absent. In those reads where the phage was missing, the sequence at 2009359–2009371 was duplicated. Primers were designed to the chromosome flanking this region and in contig 3 facing outwards. All combinations of primers amplified, supporting the conclusion that contig 3 is a phage that exists in both integrated and circular forms at this locus (Additional file 1: Figure S1).

The chromosome contains a region containing 39 CRISPR repeats along with 8 CRISPR-associated genes. The CRISPR spacers were aligned with BLAST against the genome and two of them were found to match the two putative phage regions. This suggests that this strain of *T. saccharolyticum* has a history of infection and defense against these two phage [28]. Analysis of *C. thermocellum* also showed possible prophages and much more numerous and extensive CRISPR repeats and CRISPR-associated genes, possibly related to the low transformation efficiency of *C. thermocellum* [29]. Additional analysis across other Clostridia show further CRISPR features [30].

A high percentage of genes (11.2%) have predicted functions (i.e. COG category) related to carbohydrate transport and metabolism. For comparison, only 6.5% of the ORFs in *C. thermocellum* ATCC27405 are assigned to this functional group. Both ABC-type and phospotransferase transporters occur. The tool dbCAN [31] was used to compare all *T. saccharolyticum* protein sequences to hidden Markov models (HMMs) of all protein families in the CAZY database. The program identified 73 ORFs with similarity to glycosyl hydrolase HMMs, including 3 in glycosyl hydrolase family 5 with a predicted function of “cellulase,” all of which had at least one match with an E-value equal to or better than 0.01 (with all but one being better than 0.001). It also identified 18 proteins with similarity to Cellulose Binding Module HMMs. It should be noted though that *T. saccharolyticum* does not grow on crystalline cellulose such as avicel [10].

Surprisingly, a total of 67 sporulation-associated genes were identified, including *spo0A*, but the strain is sporulation deficient, although some related strains have been observed to sporulate, namely *Thermoanaerobacterium thermosaccharolyticum* and members of the genus *Clostridium* [32]. As with *Thermoanaerobacterium thermosaccharolyticum* [33]*, T. saccharolyticum* contains the nitrogenase genes required for nitrogen fixation. Sequenced members of the related genus *Thermoanaerobacter* apparently do not.

**Figure 1:**
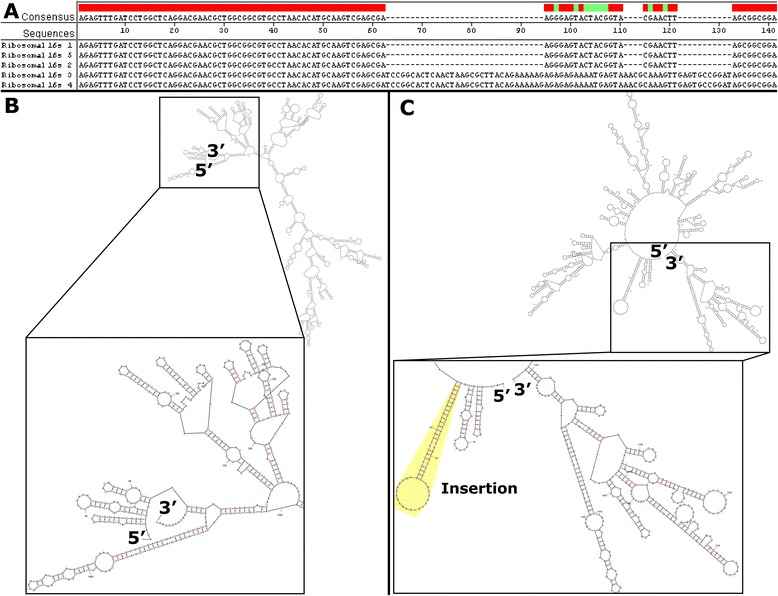
**A comparison between the two versions of the 16 s mRNA found in** ***T. saccharolyticum.*** **A**) an alignment and consensus sequence for a heterogeneous segment of the five 16S ribosomal components found in *T. saccharolyticum.* **B**) Mfold prediction of the structure of the shorter 16S mRNA [66]. **C**) Mfold prediction of the structure of the longer 16S mRNA.

The genome contains 5 ribosomal regions, all oriented in the same direction. Remarkably, the ribosomal sequences are not uniform, but rather of two types showing only 95% identity in the “universal” region of the 16 s subunit (Figure 1). Similar, but less pronounced heterogeneity of ribosomal sequences has been noted in other firmicutes [34], but has yet to be explained.

It is possible that these additional sequences confer some advantage during growth at elevated temperatures. Another possibility is that these modifications decrease sensitivity to an environmentally prevalent antibiotic that targets the 16 s rRNA. The 16 s rRNA is a common target for antibiotic compounds, for example aminoglycosides [35]. That said, resistance-conferring mutations are frequently single base pair changes rather than large insertion events [35,36]. In addition, at least for the aminoglycosides, the reported site of action is the A site near the 3’ end of the 16 s rRNA [36,37], whereas these insertions are very close to the 5’ end. However, the version that contains the inserts causes the 5’ and 3’ ends to no longer be located near one another, as can be seen in Figure 1 panels B and C, and thus may be playing a role in resistance.

### Effects of hemicellulose extract

Spotted microarrays were used to examine the effect that biomass-derived hydrolysate and the associated inhibitors have on *T. saccharolyticum*. In an initial experiment, cells were grown in fermenters containing rich medium and a mixture of xylose and glucose to mid-logarithmic phase, whereupon the cells were “shocked” by the addition of 10% volume of hemicellulose extract (“washate”). Control fermentations were conducted by shocking the cells with a mix of xylose and acetate at the same concentration and pH. The cells continued to grow, though growth was slightly slowed. Samples were analyzed before and up to 1 hour after the shock using spotted microarrays. Each mRNA sample was measured relative to a genomic DNA control, and all log_2_ ratios given below are relative to the gDNA control [38].

**Figure 2:**
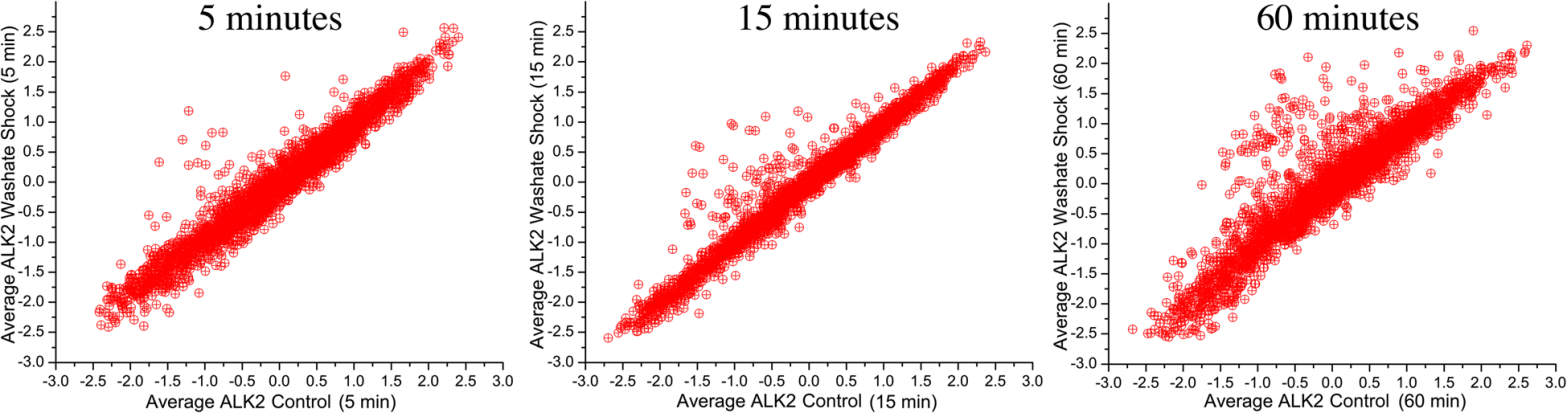
**Time points between 5 and 60 minutes post-shock with hemicellulose extract.** The horizontal axis represents log_2_ of the control xylose + acetate expression level (mRNA:gDNA ratio), while the vertical axis represents the hemicellulose extract-treated expression level. All data are the average of duplicate experiments with the exception of the 5 minutes post hemicellulose extract shock which is in triplicate.

When comparing the results from control reactors to those treated with washate, an increasing number of genes were upregulated over time in response to washate (spots above the diagonal in Figure 2). An alternate way of analyzing the same data is by comparing expression levels at a given time point to those previous to the shock (Additional file 1: Figure S2). Such comparisons versus the pre-shock culture showed more scatter, most likely due to growth phase-related gene expression changes.

**Figure 3:**
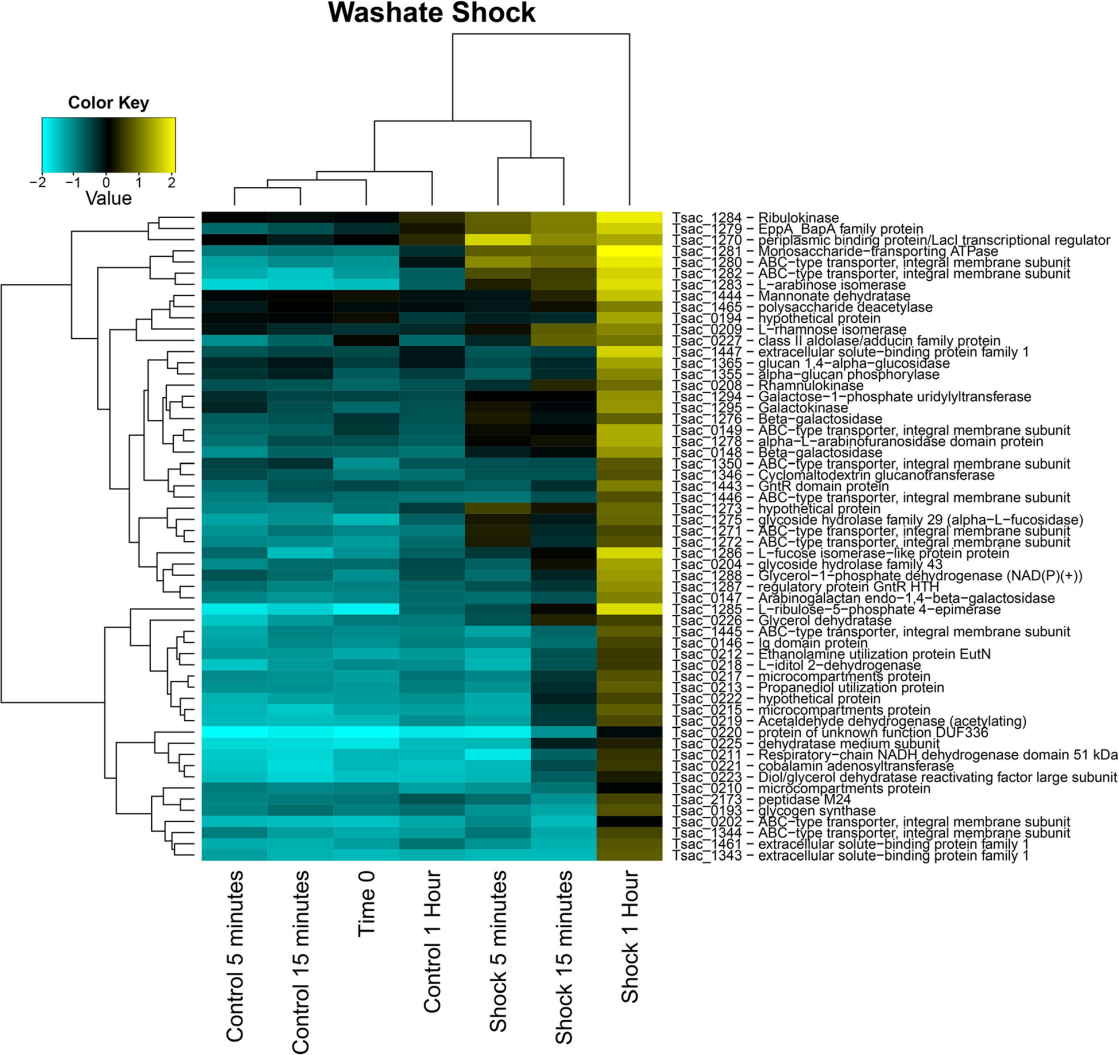
**Heat map of hierarchical clustering of genes that change in expression level upon the addition of washate with a P value of <0.01 and with a log**_**2**_ **ratio >1.0 in at least one time point**. The range of log2 mRNA:gDNA ratios is given in the color key.

Most of the genes affected by the washate were upregulated, with 58 having log_2_ ratios > 1 in at least one time point (Figure 3). At 5 min post-shock, a cluster of 17 genes (Tsac_1270-1286) was upregulated. This cluster includes glycosyl hydrolases and carbohydrate transport and catabolism genes, including three genes required for arabinose utilization. At 15 min post-shock, additional genes were upregulated, including those responsible for the formation of bacterial microcompartments and rhamnose utilization.

**Figure 4:**
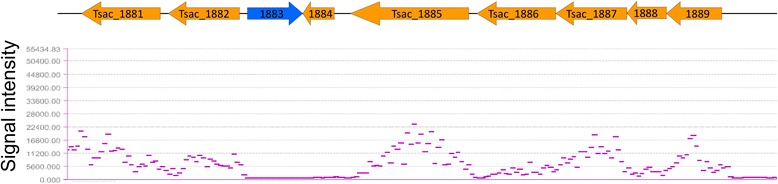
**Example of data from Nimbegen tiled microarrays (bottom) showing transcription units correlated to open reading frames (top).**

cDNA samples from before and 1 hour post washate shock were also hybridized to tiled Nimblegen microarrays. Compared to data from spotted arrays, the tiled array data showed less noise in the lower dynamic range. Moreover, by examining the expression levels visually, the boundaries of transcription units can be determined (Figure 4).

### Effects of HMF and furfural

Two of the major inhibitors in washate are furfural and hydroxymethylfurfural (HMF). To further investigate the specific effect these components have on *T. saccharolyticum*, we performed additional “shock” experiments in which HMF and furfural were added during logarithmic growth, while observing the cellular response by microarray and metabolite analysis. The levels of HMF and furfural in pretreated hardwood hydrolysates is approximately 0.1 g/L. We tested additions of HMF and furfural from 0.1 to 1.0 g/L and found that 0.5 g/L of each showed a clear effect on growth (data not shown). Notably, the effect was greatly diminished in medium containing yeast extract, so a defined medium was used in this experiment. Sample processing methods for metabolite analysis were validated as described in Additional file 1: Figure S3 and Table S1. Actively growing fermenters of *T. saccharolyticum* strain M700 at an O.D. of 0.6 were shocked with 0.5 g/L HMF and furfural. Samples were taken before the shock and at 15 minutes, 1, 2, and 4 hours after shock. These samples were analyzed via GC/MS and spotted microarrays.

**Figure 5:**
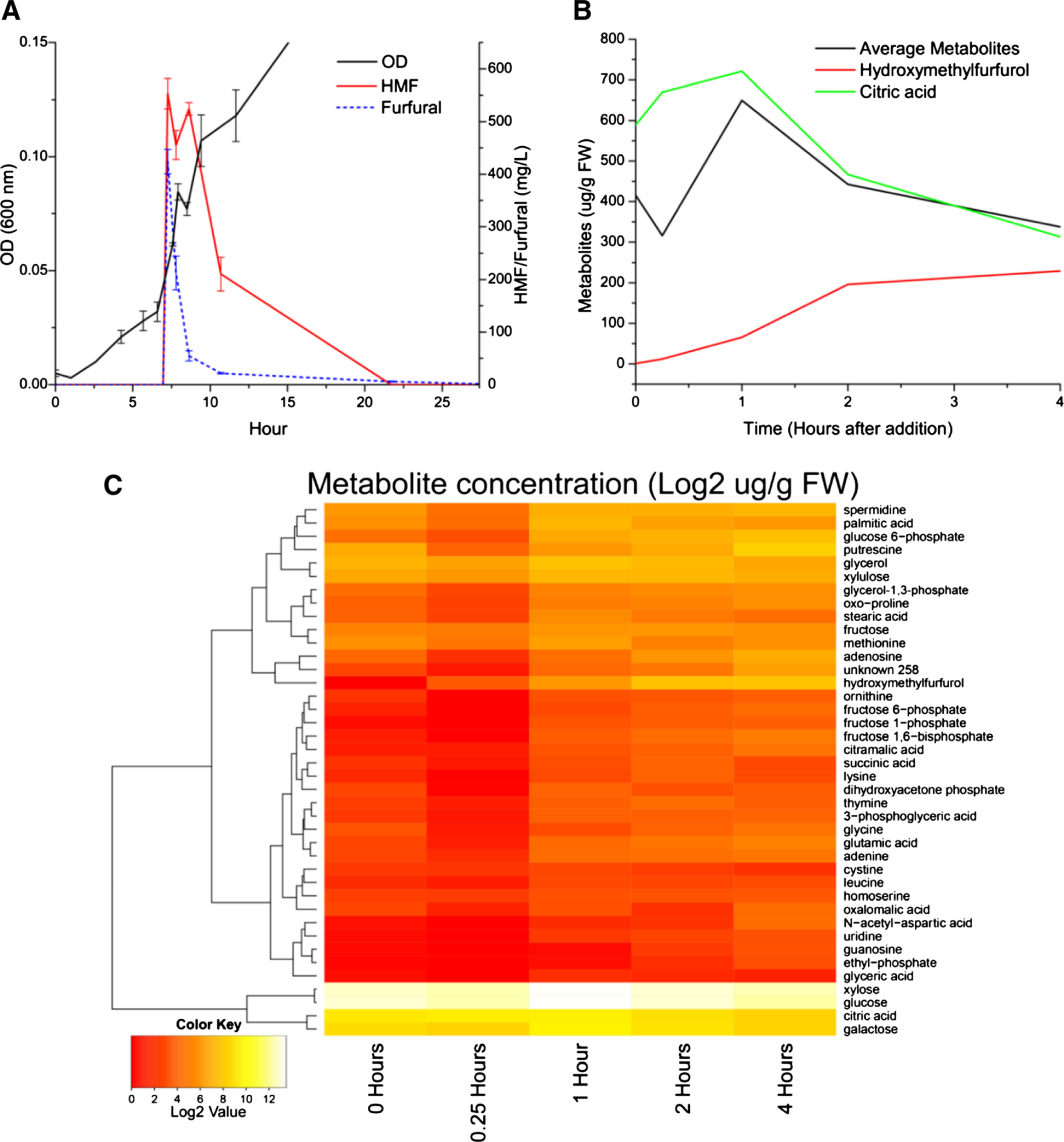
**Inhibitor shock. A**) Plot showing the addition of HMF and furfural in culture supernatants and the temporary disruption of growth. **B**) Plot showing the levels of intracellular citric acid and hydroxymethylfurfurol, as well as the average of all other metabolites. **C**) A heat map of a hierarchical clustering of the concentration of all monitored intracellular metabolites over the course of the 4 hour experiment.

A total of 40 different metabolites were tracked over the time course of the experiment (Figure 5, Additional file 1: Figure S4, Additional file 1: Table S2). Almost all metabolite concentrations showed a marked decrease at the 15 minute time point post exposure to HMF and furfural, with the exception of hydroxymethylfurfurol and citric acid. Hydroxymethylfurfurol, presumably resulting from the reduction of HMF, increased steadily throughout the 4 hours that metabolites were tracked. HMF and furfural were almost entirely metabolized after 16 hours. It is notable that glucose-6-phosphate is among the many metabolites that decrease as the result of HMF and furfural addition. This suggests that the inhibition occurs very early in the glycolysis pathway, either at glucose transport or its phosphorylation, although additional experimentation will be required to confirm this hypothesis given the labile nature of glucose-6-phosphate.

Microarray analysis of the same fermentations showed large expression differences in the phage loci between replicates during growth in the presence of HMF and furfural (Additional file 1: Figure S5). Other non-phage genes were also observed to change sympathetically with the phage genes, including the aforementioned CRISPRs.

In order to determine if some of the same genes were affected by the addition of HMF/furfural as by washate, a comparison of the two datasets was performed. The log_2_ ratio difference was calculated and analyzed via *t*-tests using the control from the same time point as reference for washate shock and using the pre-shock as reference for HMF/furfural shock. The 15 minute and 60 minute time points were considered for each, and the greater log_2_ ratio or significance value was used. In the washate shock experiment 502 genes were significantly affected (P value < 0.05) in either the 15 or 60 minute time points, and in the HMF/furfural shock experiment, 414 genes were affected in either the 15 or 60 minute time points. Between the two sets of significant genes, 88 were in common. Of these, 40 had a log_2_ difference in either experiment greater than 0.7, and 9 had a log2 difference greater than 0.7 in both (Additional file 2). Among these notable genes upregulated after both types of shock are members of a gene cluster related to sulfur assimilation (Tsac_1655-1665) possibly playing a role in sulfur utilization from expected or actual sources of sulfur present in some plant polysaccharides [39-41], alanine dehydrogenase (Tsac_2175) and NADPH-dependent methylglyoxal reductase (Tsac_1406). It should be noted, however, that this comparison is less than ideal in that different media and strains were used and that phage activity was noted in half of the HMF + furfural shock samples.

A wealth of other microarray and metabolite data were generated (Table 1). Note that at each timepoint listed in Table 1, multiple biological replicates were usually generated. In addition to testing numerous conditions, a variety of engineered and evolved strains were also analyzed. These strains were created as a part of our ongoing efforts to optimize *T. saccharolyticum* for industrial ethanol production, and are described in detail elsewhere [4,20,26,42,43]. The data are available as Additional files 2, 3, 4 and 5.

**Table 1:** **Summary of microarray and metabolomics data sets**

**Strain**	**Medium**	**Condition**	**Microarray timepoints analyzed**	**Metabolite timepoints analyzed**
ALK2	MTC	arabinose	1	
ALK2	MTC	cellobiose	2	5
ALK2	MTC	cellobiose: nitrogen-limited	4	4
ALK2	MTC	cellobiose fed-batch: N-limited	4	5
ALK2	MTC	enzymatic hydrolysate	1	
ALK2	MTC	glucose-arabinose-mannose	4	
ALK2	MTC	glucose-xylose	7	
ALK2	MTC	glucose-xylose-cellobiose	1	
ALK2	MTC	glucose-xylose-ethanol	1	
ALK2	MTC	glucose-xylose-acetate shock	3	
ALK2	MTC	glucose-xylose-washate shock	3	
ALK2	MTC	pretreated hardwood SSF	3	
ALK2	MTC	xylose fed-batch	4	5
M0355	MTC	glucose-xylose	1	
M0355	MTC	glucose-xylose-ethanol	1	
M0355	MTC	pretreated hardwood SSF	1	
M0521	MTC	pretreated hardwood SSF	2	
M0700	MTC	glucose-xylose	2	
M0700	MTC	glucose-xylose-ethanol	2	
M0700	TSD	glucose-xylose HMF + furfural shock	5	5
M1151	TSC3	cellobiose-maltodextrin	4	4
M1151	TSC4	xylose-detoxified washate shock	7	3
M1291/1442	TSC4	sigmacell SSF	2	6
M1732	TSC7	xylose-diamide shock	6	5

### Genome Scale *in silico* metabolic model

Genome-scale constraint-based metabolic models are useful tools for exploring the metabolic capabilities of an organism and for integrating bioinformatics data sets with the metabolic network. A genome-scale model for *Thermoanaerobacterium saccharolyticum* was built for this study based on its genomic content, current literature, and experimental data (Additional file 6). An initial reaction list was built by comparing its genetic content with that of the related bacterium *Clostridium thermocellum*, for which a curated metabolic model already exists [44]. To do this, a BLAST search was performed using the genes included in the *C. thermocellum* model versus the *T. saccharolyticum* ORF predictions (unidirectional, E-value cut off 0.01). This resulted in an initial set of 425 reactions with gene-reaction mappings to serve as the foundation of the reconstruction. Additionally, metabolic pathways for xylose and sorbitol metabolism were added, and cellulose breakdown reactions were removed having never been considered in the reconstruction. The bifurcating ferredoxin:NAD oxidoreductase described by Wang *et al.* was added as well [45]. A number of other changes were made based on biochemical evidence, and additional gap filling was performed as described in the Methods section to generate a working model. These changes are detailed in Additional file 7. The final model contains 528 metabolites and 516 reactions associated with 315 genes. A comparison of these statistics to the *C. thermocellum* model is shown in Table 2.

**Table 2:** **A comparison between the number of components in the models generated for** ***T. saccharolyticum*** **and** ***C. thermocellum*** **.**

	***T. saccharolyticum***	***C. thermocellum*** ^**a**^
Genes	315	432
Metabolites	503	525
Reactions	515	577
- Gene-associated	461 (90%)	463 (80%)
- Biomass	1	1
- Non-gene associated [cytosolic]	43	60
- Non-gene associated [transport]	11	54

^a^[44].

### Model validation

Although the metabolic network composition at this stage was consistent with available information based on genome annotation and experimental observations [4,5,20,24,26,42], the resulting flux space remained highly underdetermined. This is a consistent challenge facing all constraint-based models, because many thermodynamic and regulatory effects cannot be captured in the stoichiometric network. In particular for the *T. saccharolyticum* model, the diversity of hydrogenase systems hosted by this organism, left unconstrained, provide the network with many ways to efficiently regenerate cofactors, allowing biologically unrealistic levels of flux towards acetate and hydrogen. From a thermodynamic standpoint, actual allowable fluxes through these reactions are limited by many factors, including the intracellular concentrations of the cofactors, the concentration of hydrogen, intra- and extracellular pH, and the reduction potential of ferredoxin. This problem is complicated further by the kinetics and expression levels of the responsible enzymes. In the absence of the necessary parameters to formulate these constraints, we decided on a top-down approach to replicate experimental observations by making some of the hydrogenase reactions irreversible and by limiting the overall hydrogen production to observed yields. In a previous study [42], the four gene operon *hfs* coding for the reaction ferredoxin hydrogenase was found to be the primary hydrogen producer *in vivo*, whereas the other hydrogenase genes tested were found to contribute only slightly or not at all to hydrogen production. Reflecting this in the model, the energy-conserving hydrogenase (ECH) was blocked, and the bifurcating hydrogenase (BIFH2) and the NADH hydrogenase (NADH2) were forced to be irreversible in the direction of hydrogen uptake. Additionally, total hydrogen export was limited to a yield of 0.9 M H_2_:M glucose to reflect the *in vivo* measurements [42]. These modifications had a dramatic impact on the predicted performance of the model by limiting the amount of reducing equivalents that could be sent to hydrogen production, thereby shifting some carbon flux from acetate to ethanol and other organic acids. Further experimentation with hydrogenase constraints may prove useful to help understand how electron and carbon flow are related in this and other mixed-acid fermentors.

**Figure 6:**
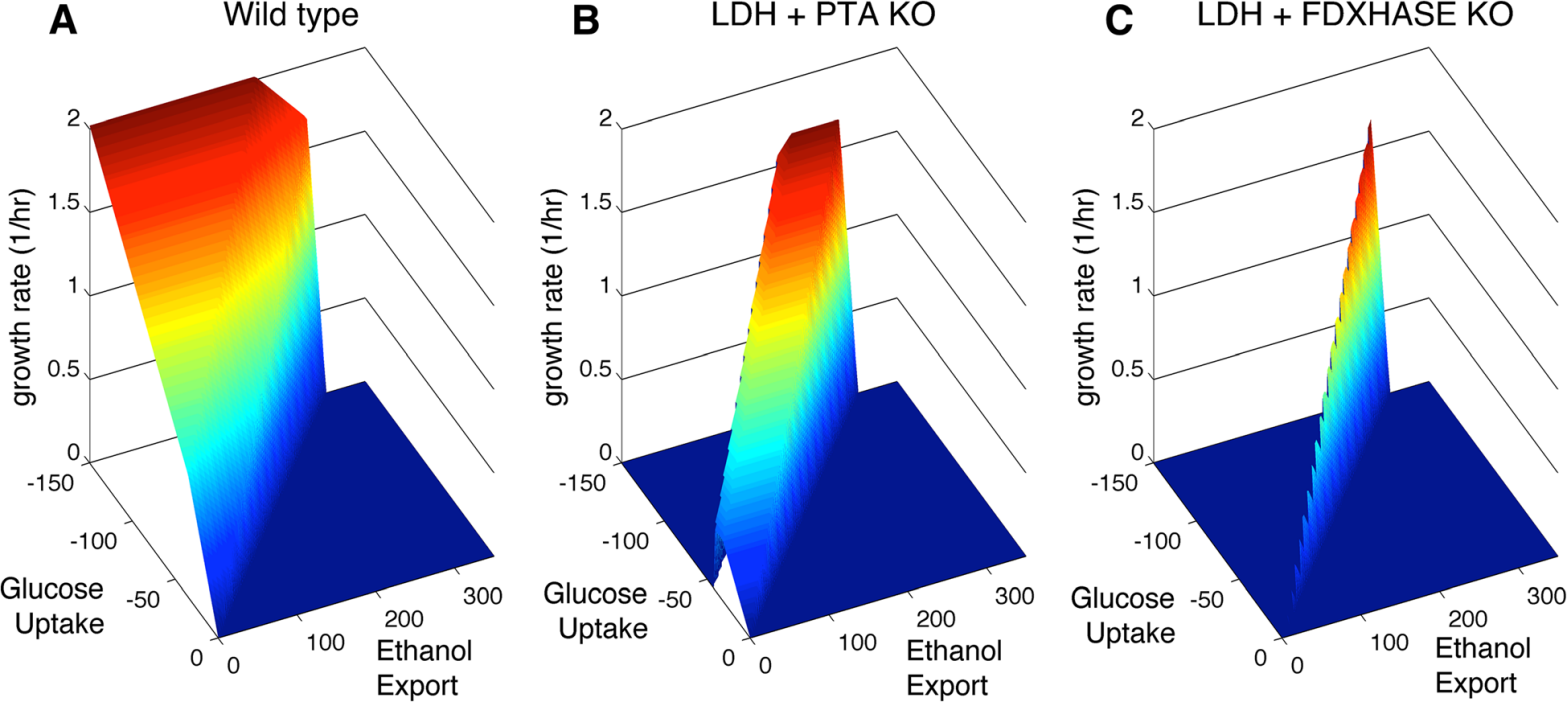
**Phenotypic phase planes for** ***T. saccharolyticum*** **high-ethanol knock out strains.** The maximum growth rate is shown as a surface over a range of fluxes for glucose uptake and ethanol production. The wild-type surface (**A**) shows the maximum growth rate occurring equally across a wide range of ethanol production rates, while the phase planes for the Δ*ldh*-*pta* strain (**B**) and the *Δldh*-*hfs* strain (**C**) demonstrate that the potential solution space is trimmed in a way that couples maximum growth to high ethanol yield.

Previous metabolic engineering efforts on *T. saccharolyticum* [4,42] have explored two distinct strategies for improving ethanol yield: a carbon-centric approach that focuses on eliminating competing carbon fluxes at the pyruvate branch point, and an electron-centric approach that disrupts the cell’s ability to produce hydrogen as a highly-reduced electron acceptor. Each of these strategies was shown to improve ethanol production to varying degrees. A phenotypic phase plane analysis was performed to explore the metabolic implications of these knockout strategies. Figure 6 shows the optimal growth surfaces for these knockouts over the complete ranges of possible carbon uptake and ethanol production rates. In the wild-type seen in Figure 6-A, optimal growth can occur across a wide range of ethanol flux values, limited by the maximum glucose uptake rate. Knocking out the lactate dehydrogenase (LDH) and phosphotransacetylase (PTA) reactions eliminates stoichiometrically equivalent solutions, leading to a maximum growth rate that is coupled to high ethanol production (Figure 6-B). The coupling of ethanol flux to growth rate was found to be much stronger, however, in the electron-centered strategy (Figure 6-C), which removed the reactions for LDH and ferredoxin hydrogenase (HFS). This knockout strategy greatly limits the available solution space and tightly dictates the ethanol yield at some penalty to the maximum growth rate. This finding is consistent with experimental results, which found a lower overall growth rate and cellobiose uptake rate in the *ldh-hfs* deletion strain when compared to the wild-type or *ldh-pta-ack* deletion strain [42]. However, the strong coupling of ethanol production to growth rate in the *ldh-hfs* knockout strain implies that it may be a good candidate for adaptive evolution to improve ethanol productivity.

**Figure 7:**
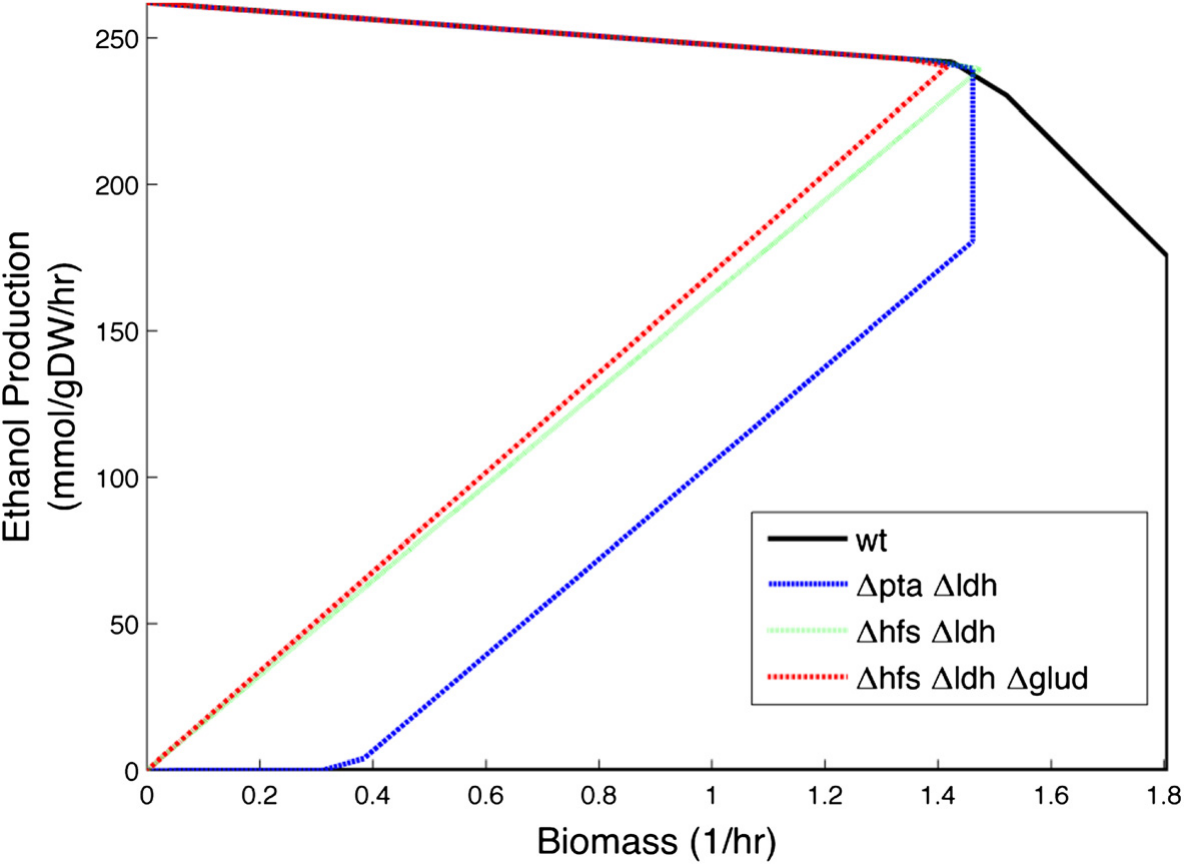
**Growth envelope for various ethanol strain designs during growth on glucose.** ΔLDH-ΔHFS and ΔHFS-ΔLDH-ΔGLUD were both identified by OptKnock as being optimal designs for ethanol production.

We attempted to determine if any other knockout strain designs would maximize ethanol production at an optimal growth rate. The bilevel optimization algorithm OptKnock [46] was used to search for knockout strain designs that would improve production of ethanol by coupling it to improved growth rate. When OptKnock searches a maximum of 2 reaction knockouts, optimal ethanol production is predicted when knocking out LDH and HFS. When allowing three reactions knockouts, OptKnock finds a marginal improvement by deleting LDH, HFS, and glutamate dehydrogenase (GLUD). Removal of GLUD forces the cell to use the reactions glutamate synthase (GLUS) and glutamine synthetase (GLNS) in order to incorporate ammonium, spending an additional mole of ATP per mole of ammonium (Table 3). This inefficiency predicts only a marginal improvement in ethanol production of 0.3% over the ∆LDH-∆HFS strain (Figure 7).

**Table 3:** **Relevant reactions in ethanol producing knockout strain designs**

**ID**	**Reaction Name**	**Formula**	**Gene Association**
**GLNS**	Glutamine synthetase	glu-L[c] + ATP[c] + NH4[c] - > ADP[c] + Pi[c] + H[c] + gln-L[c]	Tsac_2029
**GLUDy**	Glutamate dehydrogenase (NADP)	NADP[c] + H2O[c] + glu-L[c] < = > H[c] + NADPH[c] + NH4[c] + akg[c]	Tsac_2172
**GLUSy**	Glutamate synthase(NADPH)	H[c] + NADPH[c] + gln-L[c] + akg[c] - > NADP[c] + 2 glu-L[c]	Tsac_1234
**LDH_L**	L-lactate dehydrogenase	lac-L[c] + NAD[c] < = > NADH [c] + H[c] + pyr[c]	Tsac_0179
**PTAr**	Phosphotransacetylase	Pi[c] + AcCoA[c] < = > CoA[c] + actp[c]	Tsac_1744
**HFS**	Ferredoxin hydrogenase	Fdred[c] + 2 h[c] < == > Fdox + H2[c]	Tsac_1550 & Tsac_1551 & Tsac_1552 & Tsac_1553

## Conclusions

Here we report the first genome scale study of the industrially important bacterium *T. saccharolyticum*. This work informs and supports not only the study of fundamental microbial physiology, but also its potential applications in this organism. The resources presented herein will facilitate further efforts to engineer *T. saccharolyticum* for the production of biofuels. In addition, ongoing engineering efforts in other organisms to increase inhibitor tolerance and ethanol yield and titer may benefit from these data.

## Methods

### Strains

The tiled microarrays were designed based on wild-type *T. saccharolyticum* YS485 DSM 8691 (Deutsche Sammlung von Mikroorganismen und Zellkulturen GmbH, Germany). The microarrays and metabolite profiling were performed using engineered and evolved ethanologenic strains of *T. saccharolyticum*, described previously [26].

### Growth media

MTC media [47] included vitamins and trace minerals as described, plus 10 g/L yeast extract and 5 g/L Difco Tryptone. It was supplemented with various concentrations of glucose, xylose, and mixtures of hemicellulose extract or acetic acid mixed with xylose. These concentrations are noted for each experiment. TS5 media was developed specifically for *T. saccharolyticum*. It is similar to the previously published media TSC1 [20] but with only 0.5 g/l KH_2_PO_4_ and with 0.5 g/l tryptone. The full media formulation per liter is: Solution I (yeast extract 8.5 g, 1.85 (NH_4_)_2_SO_4_, 0.05 g FeSO_4_, 0.5 g KH_2_PO_4_, 1 g MgCl_2_ * 6 H2O, 0.05 g CaCl_2_, 0.5 g Tryptone, 2 g Trisodium citrate * 2 H_2_O, 800 ml H_2_O) and Solution II (10 g Xylose, 200 ml H_2_O). These are autoclaved separately to avoid caramelizing the xylose, then mixed.

### Hemicellulose extract

Hemicellulose extract, or ‘washate’, for the microarray and metabolite profiling experiments was prepared by suspending mixed hardwood pretreated with steam in an Andritz horiontal plug-flow reactor to severity 3.8 in water at 30% solids. It was then autoclaved for one hour, and the liquid was removed from the solids by vacuum filtration using Whatman Grade No. 1 Filter Paper (Whatman Ltd, Kent, UK). It was then brought to pH 6.0 using NH_4_OH. The extract contained 11.52 g/L monomeric xylose, 0.89 g/L glucose, 0.84 g/L lactate, 3.54 g/L acetate, 0.56 g/L HMF, 0.26 g/L furfural, and various other inhibitors. Additional carbohydrate was present but not analyzed due to its oligomeric state or lack of standards for analysis.

### Genome sequencing of T. saccharolyticum YS485

The genome of *T. saccharolyticum* JW/SL-YS485 was generated over a 4 year span by a variety of techniques. Initially, a clone library was constructed and Sanger sequenced to 8× coverage. Clones were selected for additional sequencing to close gaps, and additional sequence data was generated with the 454 platform. The assembled draft was then aligned to the complete genomes of *T. tengcongensis* and *T. pseudoethanolicus*, allowing the contigs to be ordered and oriented to each other. PCR primers were designed at the ends of contigs and used to amplify across gaps, consisting mostly of ribosomal regions. These PCR products were Sanger sequenced and used to manually close the genome. Finally, differences between the Sanger and 454 data were resolved by examining sequence data from various strains sequenced with Illumina technology. Genes were identified using Prodigal [48] as part of the Oak Ridge National Laboratory genome annotation pipeline, followed by a round of manual curation. The predicted CDSs were translated and used to search the National Center for Biotechnology Information (NCBI) nonredundant database, UniProt, TIGRFam, Pfam, PRIAM, KEGG, COG, and InterPro databases. These data sources were combined to assert a product description for each predicted protein. Non-coding genes and miscellaneous features were predicted using tRNAscan-SE [49], RNAmmer [50], Rfam [51], TMHMM [52], and signalP [53]. The genome sequence has been assigned GenBank accession numbers CP003184.1, CP003185.1 and CP003186.1 (the genome, the mega-pasmid, and the phage, respectively).

### Phage confirmation

Primers were designed to confirm the presence of a phage in contig 3 which is present in both integrated and circular forms (C17_near_endF: CTGCCCGTGGAACATCTAAT, C17_near_startR: GTTGGTTCTGCCCTGTTTGT, C15_int_siteF: TTTGCACCGCCATTTAAGAG, C15_int_siteR: ACGGTGATGAAGAAGCGAAA, C18_near_startR: AATTCGGCATGTGTTGGAT). PCR was performed on genomic DNA from *T. saccharolyticum* strain YS485 and *T. saccharolyticum* M700 and products were separated on a 1% agarose gel.

### Spotted microarray construction, hybridization and analysis

Spotted oligonucleotide microarrays were essentially constructed and hybridized as described previously [54,55]. Briefly, DNA sequences that represented predicted-protein encoding sequences were obtained for the *T. saccharolyticum* YS485 genome (NCBI GenBank accession numbers CP003184.1, CP003185.1 and CP003186.1) and 70-mer oligonucleotide probes were designed using the CommOligo software [56]. The original genome sequence was in draft format and 2,627 oligonucleotide probes were designed for 2,667 putative CDS, representing 98.5% of the predicted-protein encoding sequences for the draft genome. Subsequently, refinements were made as the genome sequence was closed. Oligonucleotides were commercially synthesized without modification (Integrated DNA Technologies, Coralville, Iowa) in 96-well stock plates and transferred to 384-well printing plates in a final concentration of 50% DMSO using a BioMek FX liquid handling robot (Beckman-Coulter, Fullerton, CA). Probes were then spotted onto UltraGAPS glass slides (Corning Life Sciences, Corning, NY) using a BioRobotics Microgrid II microarrayer (Genomic Solutions, Ann Arbor, MI) in a dust-free clean room maintained at 21°C and 50% relative humidity. Spotted DNA was stabilized on slides by ultraviolet cross-linking using a UV 1800 Stratalinker (Stratagene, La Jolla, CA) according to slide manufacturer’s instructions (Corning Life Sciences).

Total RNA was purified using an RNeasy Plus Mini Kit (Qiagen), which was used as template to generate cDNA copies labeled with Cy5-dUTP (Amersham Biosciences, Piscataway, NJ). Labeled genomic DNA (Cy3) was used as a control and as the common reference to co-hybridize with labeled RNA (Cy5) samples for each slide. Microarray hybridization and washing conditions have been described elsewhere [54,55,57]. Microarray images were scanned using a ScanArray Express (PerkinElmer) scanner, and spot signal, quality, and background fluorescent intensities were quantified using ImaGene version 6.0 (Biodiscovery, Marina Del Rey, CA). Outlier detection, background correction, normalizations and log ratios were generated as described previously [57], except that the workflow was conducted using JMP Genomics (SAS Institute Inc.) with custom scripts.

### Washate shock microarrays

*T. saccharolyticum* ALK2 was grown overnight shaking in bottles with 50 ml MTC + 5 g/L glucose and xylose to an optical density of 0.5 at 600 nm. 25 ml from these bottles were inoculated into 1 L of MTC media + 2.7 g/L xylose + 4.6 g/L glucose. Fermentations were performed in duplicate at 1 L volume in Sartorius BiostatA+ reactors maintained at pH 5.8, 55°C, stirring 150 rpm, and purged with N2/CO2 prior to inoculation. Upon reaching an OD of 0.6, 100 ml of either a control solution (11.5 g/L xylose, 3.5 g/L acetic acid, with pH adjusted to 6.0 with NH_4_OH) or washate was added. Samples were taken at time 0, 5, 15, and 60 minutes after shock. The samples were mixed with 30 ml RNAprotect bacteria reagent (QIAGEN Corp, Valencia, CA) and left at room temperature for 5 minutes. The samples were then centrifuged at 4000 rpm for 10 min at 4°C. The pellets were then resuspended in 1 ml SET buffer (Sucrose-EDTA-Tris buffer: 50 mM Tris–HCl, pH 8.0, 50 mM EDTA, 20% w/v Sucrose) and stored at −80°C.

### HMF and furfural shock microarrays

*T. saccharolyticum* M700 was grown overnight in bottles with 50 ml Defined TS5 media (without tryptone or yeast extract) shaking at 55°C. 25 ml from these bottles were inoculated into 4 reactors containing 1 L of Defined TS5 and maintained at pH 5.8, 55°C, stirring 150 rpm, and under constant purging with N_2_/CO_2_. The reactors were grown to an O.D. of 0.06 at which point 0.5 g/l each of HMF and furfural were added to two of the reactors, leaving the second two as controls. Samples were taken at times 0, 15 minutes, 1, 2, and 4 hours after addition. Two sets of samples were taken at each time point, one for microarrays and one for proteomics. The samples for microarray analysis were mixed with 30 ml RNAprotect bacteria reagent (QIAGEN Corp, Valencia, CA) and left at room temperature for 5 minutes. The samples were then centrifuged at 4000 rpm for 10 min at 4°C. The pellets were then resuspended in 1 ml SET buffer and stored at −80°C. The samples for the metabolite profiling assays were centrifuged at 4°C at 4000 rpm for 10 minutes, supernatants were poured off and the pellets were frozen at −80°C.

### Tiled microarrays

Tiled microarrays were performed by Nimblegen Corporation (Madison, WI).

### Metabolite profiling

Metabolites from *T. saccharolyticum* culture pellets and hydrolysates were analyzed as trimethylsilyl (TMS) derivatives by gas chromatography–mass spectrometry (GC/MS) using electron impact (EI) ionization, as described previously [58]. Briefly, aliquots of culture supernatants (50 μL to 2 mL) and sorbitol (aqueous internal standard added to yield 10 – 60 ng per μL injected) were transferred by pipette to a vial and stored at −20°C until analyzed. Microbial pellets were fast-frozen in liquid nitrogen and stored at −80°C until analyzed. Frozen pellets were weighed and added to 10 mL 80% ethanol containing sorbitol as internal standard. Cell pellets were ruptured by sonication with temperature maintained below 0°C, and cell debris separated from the extract by centrifugation at 4°C, and 2 mL were dried in a stream of N_2_ prior to silylation. The hydrolysate samples were thawed and also concentrated to dryness under a stream of N_2_. The internal standard was added to correct for subsequent differences in derivatization efficiency and changes in sample volume during heating. Dried extracts were dissolved in 500 μL of silylation–grade acetonitrile followed by the addition of 500 μL N-methyl-N-trimethylsilyltrifluoroacetamide (MSTFA) with 1% trimethylchlorosilane (TMCS) (Thermo Scientific, Bellefonte, PA), and samples then heated for 1 h at 70°C to generate TMS derivatives. After 2–3 days, 1-μL aliquots were injected into an Agilent Technologies Inc. (Santa Clara, CA) 5975C inert XL gas chromatograph-mass spectrometer, fitted with an Rtx-5MS with Integra-guard (5% diphenyl/95% dimethyl polysiloxane) 30 m × 250 μm × 0.25 μm film thickness capillary column. The standard quadrupole GC/MS was operated in the EI (70 eV) ionization mode, with 6 full-spectrum (50–650 Da) scans per second. Gas (helium) flow was 1.3 mL per minute with the injection port configured in the splitless mode. The injection port, MS Source, and MS Quad temperatures were 250°C, 230°C, and 150°C, respectively. The initial oven temperature was held at 50°C for 2 min and was programmed to increase at 20°C per min to 325°C and held for another 11 min, before cycling back to the initial conditions. A large user-created database (>1800 spectra) of mass spectral electron ionization (EI) fragmentation patterns of TMS-derivatized compounds, as well as the Wiley Registry 8th Edition combined with NIST 05 mass spectral database, were used to identify the metabolites of interest to be quantified. Peaks were reintegrated and reanalyzed using a key selected ion, characteristic m/z fragment, rather than the total ion chromatogram, to minimize integrating co-eluting metabolites. The extracted peaks of known metabolites were scaled back up to the total ion current using predetermined scaling factors. The scaling factor for the internal standard was used for unidentified metabolites. Peaks were quantified by area integration and the concentrations were normalized to the quantity of the internal standard recovered, volume of sample processed, derivatized, and injected. Three to six replicate samples were analyzed per time point, and the metabolite data were averaged at a given time point. Unidentified metabolites were denoted by their retention time as well as key mass-to-charge (m/z) ratios.

### Constraint-based modeling of Thermoanaerobacterium saccharolyticum

Initial construction of the *Thermoanaerobacterium saccharolyticum* reaction list was based on the previously published model of the closely related species *Clostridium thermocellum* [44,59]. This was accomplished by using BLAST to search for genes in *T. saccharolyticum* that were homologous to the genes represented in the *C. thermocellum* model. Further refinement to the model was done by manual curation, incorporating available biochemical and genetic information. The resulting reaction list was not yet able to produce flux through the biomass reaction using appropriate exchange boundary conditions, so additional gap filling was required. This was accomplished through the use of a novel gap filling algorithm called FBA-gap [60] which proposes a minimal set of reaction additions necessary to support biomass production. These reactions are sourced from a reaction database populated using the reaction lists of available stoichiometric models.

Flux balance analysis (FBA) [61], was used throughout the reconstruction and analysis of the *T. saccharolyticum* model to simulate optimal growth. Modeling work was done using the COBRA toolbox for Matlab [62,63] along with custom methods and the Gurobi Optimizer. OptKnock [46] was used to search for knockout strains that would putatively couple ethanol production with an improved growth rate. An implementation of OptKnock is available in the COBRA toolbox for MATLAB.

### Statistical analysis

Statistical analyses of metabolic modelling data were performed using R statistical software [64] and the package gplots [65].

### Availability of supporting data

The data sets supporting the results of this article are included within the article (and its additional files).

## Additional files

Additional file 1:
**Analysis of genes affected by both washate and HMF + furfural shock.** The log_2_ ratio difference was calculated (i.e. mRNA:gDNA log_2_ ratio of experimental sample minus mRNA:gDNA log_2_ ration of control, abbreviated as “Dif”) and analyzed via T-Tests using the control from the same time point as reference for washate shock and using the pre-shock as reference for HMF/furfural shock. The 15 minute and 60 minute time points were considered for each, and the greater log_2_ ratio or significance value was used for purposes of selecting the genes for inclusion here. Log2 ratios with absolute value > 1.0 are highlighted in red, and those genes which have an absolute difference > 0.7 in both experiments are highlighted in green. Additional file 2:
**Microarray data.** All microarray data was collated and normalized as one dataset using JMP Genomics. The log_2_ ratio of mRNA:gDNA is given. In the first tab, the data for individual replicates is given and in the second tab the average of replicates for each condition is given. The conditions are described in Additional file 3. Additional file 3:
**Microarray conditions.** The condition and replicate numbers correspond to those for the microarray data in Additional file 2, and information about each condition / replicate is given here. The “Sample” column gives the MP pellet number of the cells that were used to prepare the mRNA for that replicate. A textual description of the preparation of each MP pellet is given in Additional file 4. Additional file 4:
**Growth conditions and sample processing of cells pellets.** A textual description of the growth conditions for each “MP” numbered cell pellet is given. The MP numbers correspond to those described in tabular format in Additional file 3. Additional file 5:
**Metabolite measurements, given in ug/g FW (i.e. micrograms of metabolite sorbitol equivalents per g of fresh cell weight).** The experiment name, MP pellet numbers and timepoint/condition are given at the head of each column. The average of all replicates is given. Each MP number represents a biological replicate and the MP numbers correspond to descriptions given in Additional file 4. Additional file 6:
**Excel format of the metabolic reconstruction of** ***T.saccharolyticum.*** Individual tabs show the reaction list, metabolite list, changes made from the *C.thermocellum* model in developing this model, and some sample flux distributions from Flux Balance Analysis. Additional file 7:
**Metabolic reconstruction of** ***T.saccharolyticum*** **in Systems Biology Markup Language format.** The model can be visualized with tools such as CellDesigner (http://celldesigner.org/). Accessed 13 July 2015.

## Abbreviations

HMF: Hydroxymethyl furfural
ORF: Open reading frame
CRISPR: Clustered regularly interspaced short palindromic repeats
HMM: Hidden markov model
ECH: Energy-conserving hydrogenase
BIFH2: Bifurcating hydrogenase
NADH2: NADH hydrogenase
LDH: Lactate dehydrogenase
PTA: Phosphotransacetylase
HFS: Ferredoxin hydrogenase
GLUD: Glutamate dehydrogenase
GLUS: Glutamate synthase
SET: Sucrose-EDTA-tris buffer
GC/MS: Gas chromatography–mass spectrometry
MSTFA: N-methyl-N-trimethylsilyltrifluoroacetamide
BLAST: Basic local alignment search tool
FBA: Flux balance analysis
