# Supplementary material for: Genome-scale resources for Thermoanaerobacterium saccharolyticum
Source: BMC Syst Biol. 2015 Jun 26;9:30. doi: 10.1186/s12918-015-0159-x (PMC4518999; doi:10.1186/s12918-015-0159-x)
Supplement: Additional file 1: — Analysis of genes affected by both washate and HMF + furfural shock. The log2 ratio difference was calculated (i.e. mRNA:gDNA log2 ratio of experimental sample minus mRNA:gDNA log2 ration of control, abbreviated as “Dif”) and analyzed via T-Tests using the control from the same time point as reference for washate shock and using the pre-shock as reference for HMF/furfural shock. The 15 minute and 60 minute time points were considered for each, and the greater log2 ratio or significance value was used for purposes of selecting the genes for inclusion here. Log2 ratios with absolute value > 1.0 are highlighted in red, and those genes which have an absolute difference > 0.7 in both experiments are highlighted in green. [file 12918_2015_159_MOESM1_ESM.docx]

**Additional file 1: Figure S1.** PCRs showing the putative phage exists in circular and integrated forms. Also shown is the ability to span the putative integration site for the phage, showing that it is not always occupied. LANES: 1. Primers: C17_near_endF+ C17_near_startR (DNA: DSMZ YS485) 2. Primers: C15_int_siteF+ C15_int_siteR (DNA: DSMZ YS485) 3. Primers: C15_int_siteF+ C15_int_siteR (DNA: M700) 4. Primers: C15_int_siteF+ C18_near_startR (DNA: DSMZ YS485) 5. Primers: C15_int_siteF+ C18_near_startR (DNA: M700) 6. Primers: C15_int_siteR+ C18_near_startR (DNA: DSMZ YS485) 7. Primers: C15_int_siteR+ C18_near_startR (DNA: M700).

**Additional file 1: Figure S1**


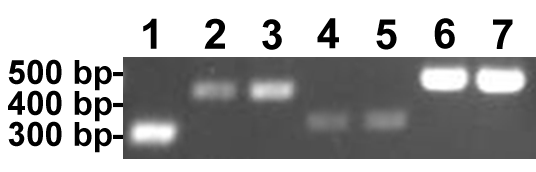


**Additional file 1: Figure S2.** Time points between 5 and 60 minutes post hemicellulose extract shock. The X axis represents log2 of the pre-shock expression level, while the Y axis represents the hemicellulose extract treated expression level. All data are the average of duplicate experiments with the exception of the 5 minutes post hemicellulose extract shock which is in triplicate.

**Additional file 1: Figure S2**


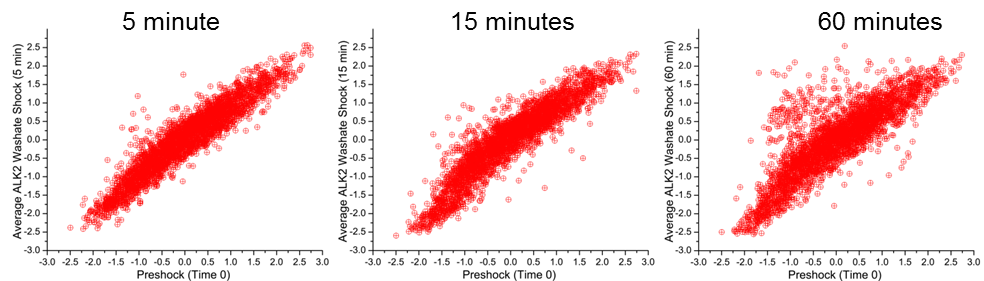


**Additional file 1: Figure S3.** Sample processing validation. A) Growth data: *T. saccharolyticum* strain ALK2 was grown in 1 L of MTC medium with 8.5 g/L yeast extract at pH 5.5 at 55°C. One of two replicate cultures is shown. 30 ml samples were removed at times indicated with arrows. The samples were split into 3 and processed either by centrifugation for 5 min in a chilled centrifuge, or centrifuged then washed in 1 ml chilled 5% sucrose and centrifuged again, or mixed with 2 volumes of chilled 60% methanol + 10 mM ammonium acetate and centrifuged. Pellets generated by all 3 methods were then resuspended in 1 ml chilled 5% sucrose and stored at -80°C until extraction and analysis by GC/MS. Note that sucrose was found to interfere with derivitization chemistry, likely by competing for trimethylsilyl groups, though the internal standard recovery was good. Resuspension in sucrose was later eliminated from our preferred method. B-E) Metabolite levels in extracted cell pellets as measured by GC/MS. The recovery of all four metabolites shown was much higher when cells were processed by simple centrifugation. Washing and methanol extraction likely led to leakage of metabolites from the cell (Canelas AB et al, Metabolomics 2008, 4:226).

**Additional file 1: Figure S3**


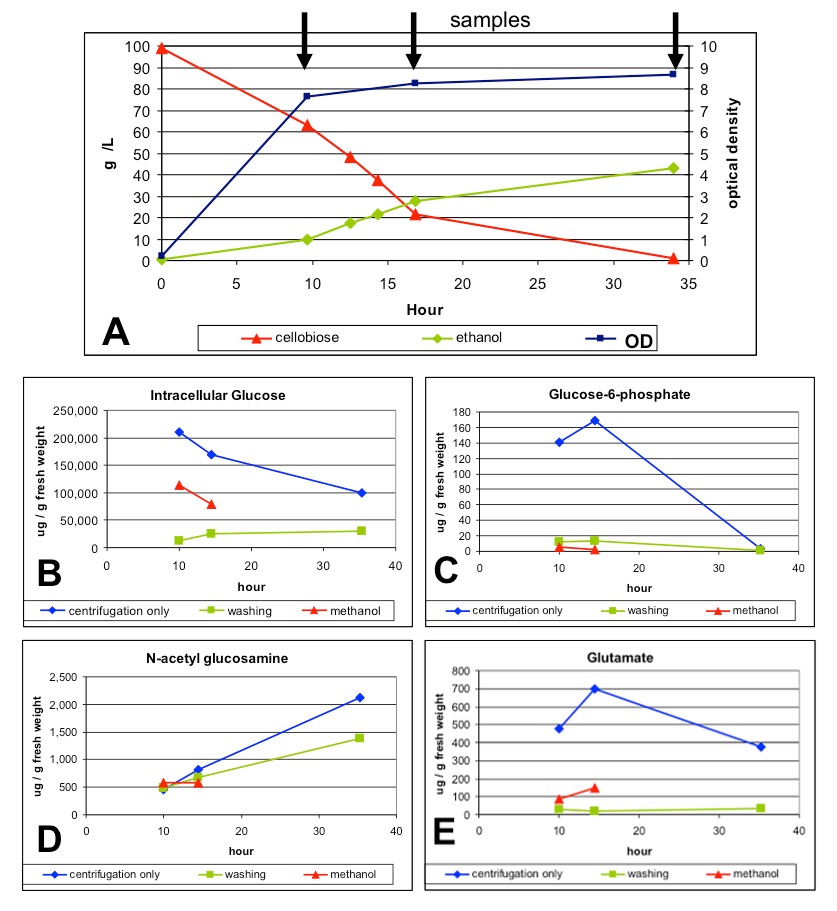


**Additional file 1: Figure S4.** Averages of metabolites from HMF/furfural shock experiment between 0 hours before addition and 4 hours after.

**Additional file 1: Figure S4.**


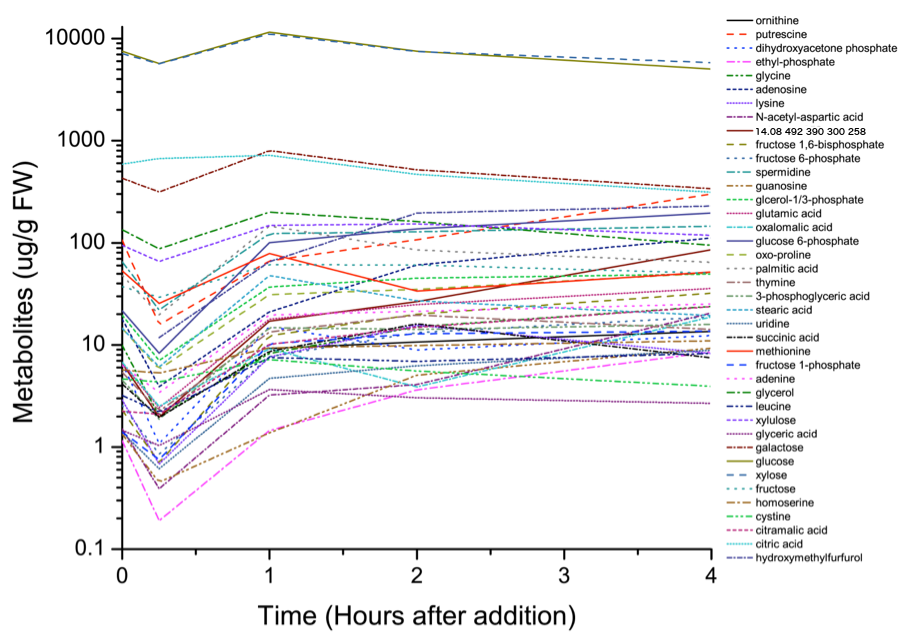


**Additional file 1: Figure S5.** A heat map showing the inter-replicate differences between phage expression in two reactors. Top 25 genes were chosen based on averages of absolute differences between replicate reactors. The mean inter-replicate expression differences for the displayed genes are 1.76 – 2.90 (log10 values). For the data set as a whole the mean inter-replicate expression difference is 0.33 and the mode is 0.24 (log10 values). All but one of these genes (Tsac_2567) are phage related.

**Additional file 1: Figure S5.**


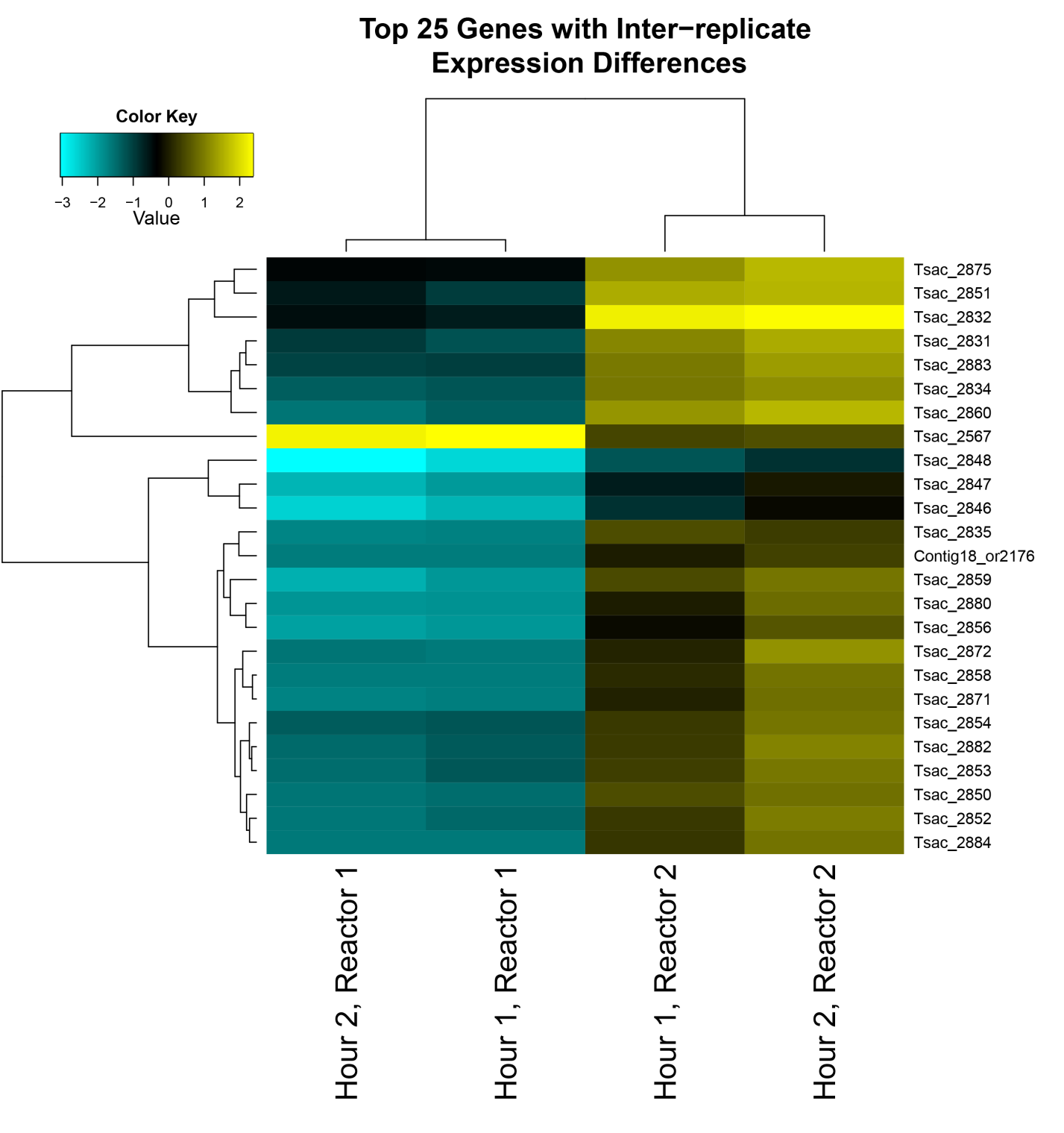


**Additional file 1: Table S1.** Metabolite carryover experiment. In order to evaluate whether metabolite levels such as glucose could be the result of carryover from the growth medium, an experiment was conducted in which a non-metabolized sugar was added to the medium before sample processing, then measured in the cell pellet by GC/MS. First, ribose and melibiose were determined to be unable to support growth of *T. saccharolyticum* in rich medium. Then strain ALK2 was grown in MTC medium + 8.5 g/L yeast extract + 10 g/L glucose to an optical density of 0.7. Samples were removed into tubes containing either ribose or melibiose at a final concentration of 10 g/L, then centrifuged and processed for metabolite analysis. Control samples with neither added sugar were also processed. As expected, the control showed neither added sugar by GC/MS. Measured levels of ribose and melibiose are indicated in the table, along with calculations that show that carryover was less than 1% of the maximum that could be expected if the entire volume of cell pellet was growth medium.

**Additional file 1: Table S1.**

**Additional file 1: Table 2.** The average concentration of each metabolite at 0, 0.25, 1, 2, and 4 hours after HMF/Furfural addition.

**Additional file 1: Table 2**

|  | Metabolite concentration in ug/g FW | | | | |
| --- | --- | --- | --- | --- | --- |
| Metabolite | 0 Hours | 0.25 Hours | 1 Hours | 2 Hours | 4 Hours |
| Ornithine | 4.1 | 0 | 9.27 | 10.66 | 13.52 |
| putrescine | 106.6 | 15.8 | 66.46 | 107.27 | 301.48 |
| dihydroxyacetone phosphate | 6.63 | 1.08 | 15.03 | 8.97 | 12.34 |
| ethyl-phosphate | 1.15 | 0.19 | 1.45 | 3.63 | 8.54 |
| Glycine | 9.96 | 2.02 | 8.24 | 15.1 | 23.91 |
| adenosine | 17.91 | 3.91 | 21.07 | 60.86 | 111.83 |
| Lysine | 2.9 | 0.68 | 7.61 | 15.6 | 8.24 |
| N-acetyl-aspartic acid | 1.51 | 0.39 | 3.24 | 4.1 | 20.57 |
| 14.08 492 390 300 258 | 6.6 | 1.97 | 17.06 | 26.58 | 85.67 |
| fructose 1,6-bisphosphate | 2.31 | 0.7 | 12.32 | 20.01 | 32.13 |
| fructose 6-phosphate | 2.56 | 0.85 | 7.67 | 13.19 | 18.89 |
| spermidine | 65.49 | 22.04 | 122.96 | 128.28 | 145.03 |
| guanosine | 1.33 | 0.45 | 1.38 | 5.07 | 9.32 |
| glycerol-1,3-phosphate | 19.52 | 7.05 | 37.02 | 45.02 | 49.5 |
| Glutamic acid | 6.62 | 2.43 | 17.95 | 24.91 | 35.87 |
| oxalomalic acid | 6.76 | 2.51 | 9.19 | 3.88 | 18.81 |
| glucose 6-phosphate | 21.65 | 8.34 | 100.15 | 137.14 | 195.63 |
| oxo-proline | 14.6 | 5.8 | 31.18 | 35.02 | 50.81 |
| palmitic acid | 48.27 | 19.71 | 146.41 | 84.95 | 64.62 |
| Thymine | 5.4 | 2.24 | 13.39 | 19.61 | 14.18 |
| 3-phosphoglyceric acid | 4.52 | 1.88 | 14.75 | 14.2 | 15.9 |
| stearic acid | 14.59 | 6.08 | 47.98 | 27.11 | 19.39 |
| Uridine | 1.4 | 0.61 | 4.72 | 6.26 | 8.88 |
| succinic acid | 4.15 | 1.95 | 8.63 | 16.11 | 7.46 |
| methionine | 53.31 | 25.55 | 78.48 | 33.84 | 51.83 |
| fructose 1-phosphate | 1.41 | 0.75 | 10.3 | 12.82 | 13.63 |
| Adenine | 6.29 | 3.44 | 19.6 | 21.36 | 25.16 |
| Glycerol | 134.46 | 87.67 | 199.97 | 161.62 | 94.69 |
| Leucine | 3.22 | 2.21 | 7.52 | 6.89 | 8.27 |
| Xylulose | 94.92 | 65.97 | 148.56 | 153.73 | 118.08 |
| glyceric acid | 1.46 | 1.03 | 3.66 | 3.04 | 2.68 |
| galactose | 428.51 | 315.55 | 799.47 | 521.87 | 337.61 |
| Glucose | 7514.78 | 5706.81 | 11538.12 | 7521.57 | 5018.31 |
| Xylose | 7130.55 | 5648.37 | 11073.24 | 7445.69 | 5790.65 |
| Fructose | 37.16 | 29.47 | 61.33 | 60.73 | 50.48 |
| homoserine | 5.71 | 5.29 | 9.34 | 9.9 | 11 |
| Cystine | 4.66 | 4.36 | 7.19 | 5.55 | 3.93 |
| citramalic acid | 2.24 | 2.11 | 10.15 | 14.92 | 23.83 |
| citric acid | 588.82 | 669.73 | 720.92 | 466.77 | 313.1 |
| hydroxymethylfurfurol | 0 | 11.81 | 65.44 | 196.14 | 229 |
